# A survey on Canadian pediatric hospital clinical/medical teaching unit implementation during the first and second wave of the COVID-19 pandemic

**DOI:** 10.1186/s12909-021-02994-0

**Published:** 2021-11-11

**Authors:** Kara K. Tsang, Andrew Latchman, Nishma Singhal, Giuliana Federici, Sandra Russell, Denise Irwin, Robyn Stevens, Andrew G. McArthur, Sarah Khan

**Affiliations:** 1 Department of Biochemistry and Biomedical Sciences, McMaster University, Hamilton, Canada; 2 Department of Pediatrics, McMaster University, Hamilton, Ontario Canada; 3 Department of Medicine, McMaster University, Hamilton, Ontario Canada; 4 McMaster Children’s Hospital, Hamilton, Ontario Canada

**Keywords:** Rounds, COVID-19, Clinical teaching unit, Teaching, Education

## Abstract

**Background:**

As the COVID-19 pandemic heightened, infection control and prevention experts recommended clinical training opportunities be modified or discontinued, substantially impacting the function of clinical or medical teaching units (CTU). A CTU is structured to involve medical learners such that they become active participants of the health care team. Since a review of the literature demonstrates a paucity of data to guide pediatric CTU implementation during pandemic phases, we developed and disseminated a survey to assess Canadian practices.

**Method:**

A group of infectious disease specialists and pediatric hospitalists developed, tested, and disseminated surveys to understand CTU clinical rounding and teaching practices during the waves of the COVID-19 pandemic.

**Result:**

Our surveys demonstrate the variability in adapting rounding practices during this pandemic and highlights the opportunities to share our approaches and lessons learned to optimize learner experience and patient centered care during unprecedented times in our academic hospitals. We also show the pragmatic implementation of our new pediatric hospital CTU process that was informed by our survey results.

**Conclusion:**

Our study demonstrates the variability in adapting rounding practices during this pandemic and highlights the opportunities to share our approaches and lessons learned to optimize learner experience and patient centered care during unprecedented times in our academic hospitals.

**Supplementary Information:**

The online version contains supplementary material available at 10.1186/s12909-021-02994-0.

## Introduction

A clinical teaching unit (CTU) is structured to provide multiple levels of medical learners opportunities to develop hands-on skills and knowledge while actively participating in patient care. The goal of team rounding on pediatric CTUs is to provide family-centred care to the patient, while facilitating communication between multi-disciplinary health providers. Team/bedside rounds often includes an attending physician, multiple learners, bedside nurses, allied health professionals, the patient and their family members/essential caregivers. Sit down rounds are when a learner/team member performs their history/physical examination of a patient individually, and the clinical team later discusses the assessment and develops the treatment plan outside of the patient room/area. Typically, ‘sit-down’ rounds do not involve the patient or their caregivers directly. Team and ‘sit-down’ rounds are integral for medical education, patient care, establishing rapport between multidisciplinary staff, and assessment [1, 2]. The structure of team and ‘sit-down’ rounds on pediatric CTUs were disrupted as the COVID-19 pandemic heightened because of infection prevention and control guidelines for physical distancing, reducing gatherings, and the necessity for personal protective equipment conservation strategies [3]. In addition to practical changes to CTUs, the mental health of patients and health care providers has also been impacted due to the pandemic [4]. Some wards have even been converted into COVID specific wards to separate themselves from the rest of the department [5].

Specifically, in Canada, there were three waves of the pandemic, between the end of March - June 2020, September - February 2021, and March - June 2021, where CTU coordinators had to adapt to these changes. There were limited literature available and informal inquiries from peer centers on practices being implemented to optimize family-centered rounds and learning opportunities on Canadian pediatric CTUs. There were a few publications that focused on the transition of pediatric medical education towards a virtual format in the United States [3, 6, 7]. In a survey of pediatric resident medical education during COVID-19, residents found remote learning sessions as effective as traditional in-person sessions [7]. Synchronous video conferencing and asynchronous activities were developed for a pediatric cardiology department. A majority of learners agreed that the online learning was translatable to daily work [6]. In Canada, there was a lack of consensus and therefore an opportunity to survey the current practices of CTU rounds that can inform future recommendations or best practices.

There is a need to provide guidance to medical educators on how to safely and effectively implement clinical teaching rounds while recognizing we can anticipate expect fluctuations in epidemiology and therefore restrictions to continue. The aim of this study was to conduct an environmental survey to establish how teaching hospitals adapted their approach to CTU rounds implementation throughout different stages of the COVID-19 pandemic, to establish the threshold for returning back to “normal” rounds and some guidelines on when to pivot guidance in shifting epidemiology. We performed a literature review and developed surveys to explore how centers nationally have adapted their rounds and plans they have moving forward.

## Methods

We performed a literature search using Google Scholar and PubMed with search terms combining “clinical and medical teaching unit”, “Canada” and “COVID, SARS-CoV-2” and “clinical rounds, medical rounds, family-centered, patient-centered rounds” to identify whether there was literature available.

A group of infectious disease specialists and pediatric hospitalists developed an online survey using Google Forms from July-August 2020 to assess CTU implementation at Canadian pediatric hospitals. The goal of the survey was to determine a consensus on CTU implementation during the first wave of the COVID-19 pandemic (March - June 2020) and the threshold of returning back to “normal” rounds (Additional file 1). In the surveys, we describe different types of bedside and sit-down rounds to provide additional specificity on the types of rounds conducted. Survey drafts were developed iteratively by establishing the research questions, making survey edits, and testing the survey until all developers approved. The survey included multiple choice, multi-select, and short answer questions. Between November-December 2020 we then updated the Google survey to re-assess CTU implementation during the second wave of the pandemic and assess for any changes to the threshold of returning back to “normal” bedside rounds (Additional file 2). Our two surveys were sent to the Canadian Paediatric Society hospital committee email listserv which have 22 representatives from all medical schools in Canada. Each representative was only permitted to respond to each survey once. There was no exclusion criteria as the email listserv only includes appropriate and representative potential survey respondents. Responses were collected and extracted from the Google Forms spreadsheet. This study was approved by the Hamilton Integrated Research Ethics Board (#11381). We discuss descriptive statistics of our survey results herein.

## Results

While our literature search identified some publications on the changes to medical student teaching or specific medical speciality teaching (e.g., surgery, medical units) during COVID-19 [8–13], only one was specific to pediatric rounds [14]. There has been a recent publication regarding the transition to virtual family-centred rounds, where the authors describe their process of daily, virtual family-centered rounds at the Children’s Hospital of Eastern Ontario [15]. They briefly surveyed physician, nursing, and family satisfaction and assessed the time consumption of the virtual-family centered rounds. To our knowledge, there has not been any further publications on the impacts of COVID-19 on pediatric CTU implementation in Canada.

### First wave of the COVID-19 pandemic

In our survey during the first wave of the COVID-19 pandemic, we received nine responses (41% response rate) consisting of three pediatric community hospitals and six pediatric tertiary hospitals affiliated with the University of Ottawa (*n* = 1, 0.1%), University of Toronto (*n* = 3, 0.3%), University of British Columbia (n = 3, 0.3%), and McMaster University (*n* = 2, 0.2%). A majority (88.9%) of hospital CTU teams are responsible for 10-15 patients, and one hospital CTU team is responsible for 15-20 patients.

**Fig. 1:**
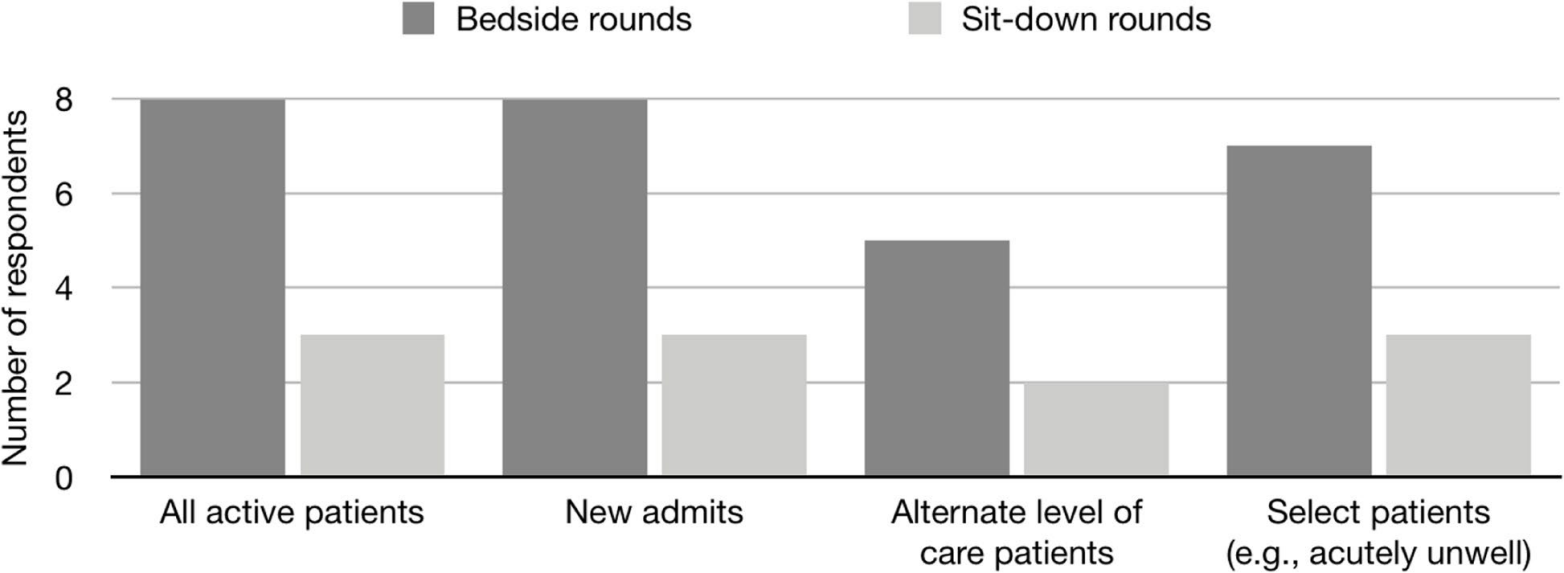
Survey question (“Before the pandemic, how were CTUs implemented at your site?”) and responses to bedside round and sit-down round implementation before the COVID-19 pandemic

To examine the changes made as a consequence of the COVID-19 pandemic, we sought to understand what standard rounding procedures existed prior to the COVID-19 pandemic. Previously all hospitals’ attendings performed daily beside rounds on all active patients and new admits and in a few (33.3%) hospitals, CTU teams also performed sit-down rounds (Fig. 1).

**Fig. 2:**
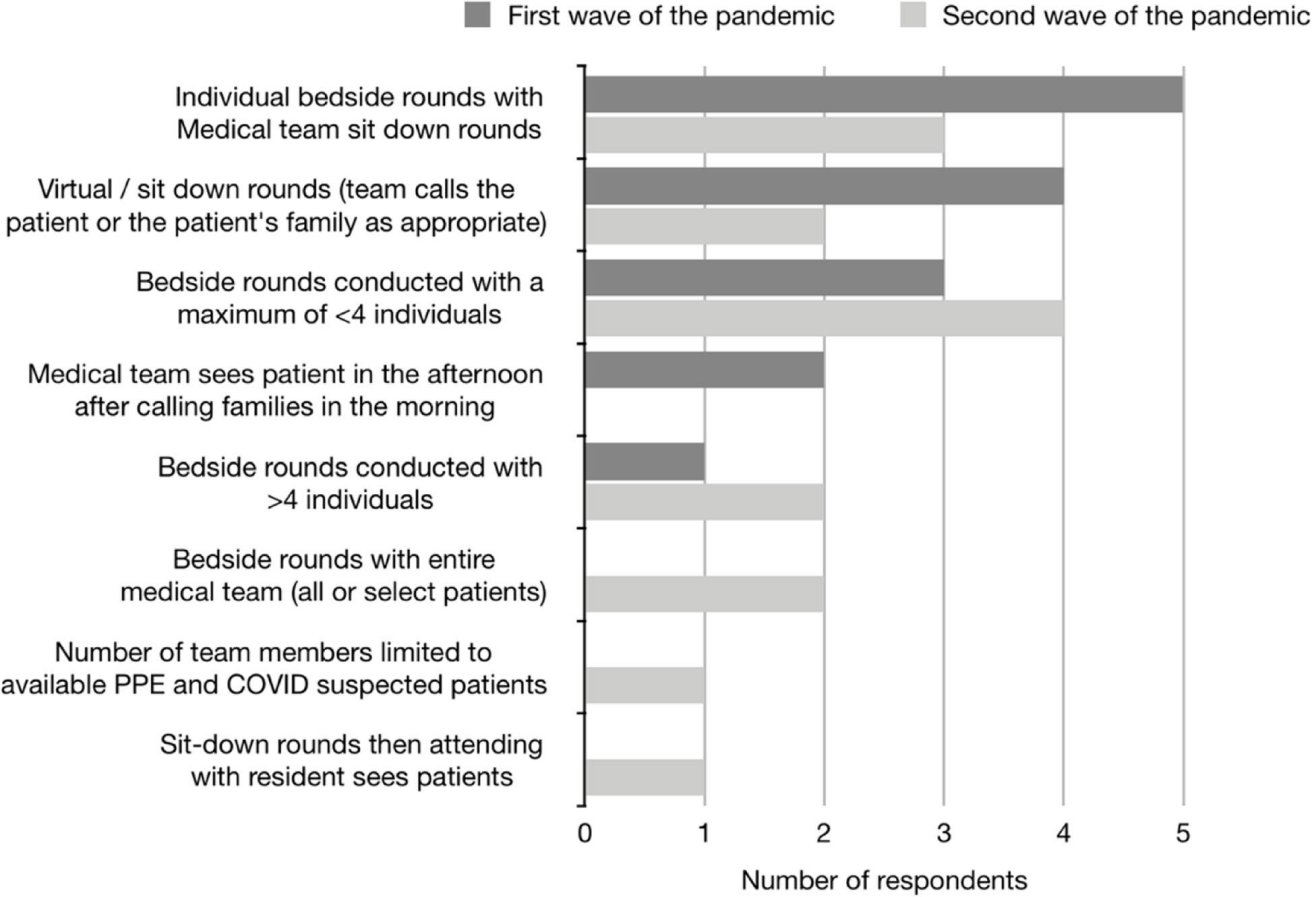
Survey question (“At the height of the first / second wave of the pandemic, what were your rounding practices?”) and responses to rounding practices at the height of the first and second wave of the pandemic

**Fig. 3:**
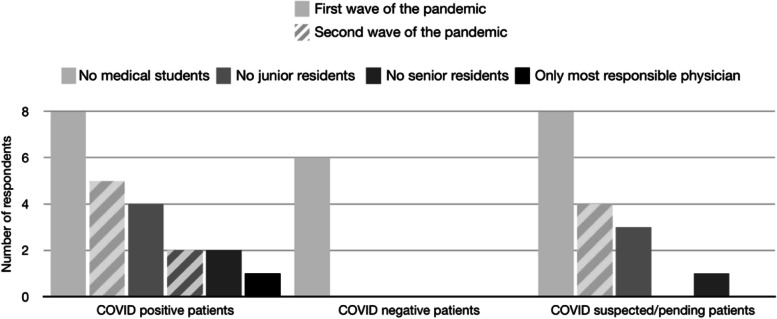
Survey question (“At the height of the first / second wave of the pandemic, which team members are allowed to participate in the care of patients?”) and responses to restrictions of team member participation in rounds during the height of the first and second wave of the pandemic

During the first wave of the COVID-19 pandemic, sit down rounds became more common (7/9 respondents) compared to before the pandemic (2/9 respondents). 44.4% (*n* = 4) of hospital CTUs utilized virtual/sit down rounds, while individual bedside rounds with the medical team were conducted on 55.6% (*n* = 5) of pediatric CTUs (Fig. 2). All medical schools (*n* = 8, 100%) restricted CTUs to not involve medical students for all COVID-19 positive/pending/suspected patients (Fig. 3). A number of CTUs also excluded junior medical residents from providing direct patient care to COVID positive (*n* = 4, 50%) or suspect patients (*n* = 3, 37.5%).

**Fig. 4:**
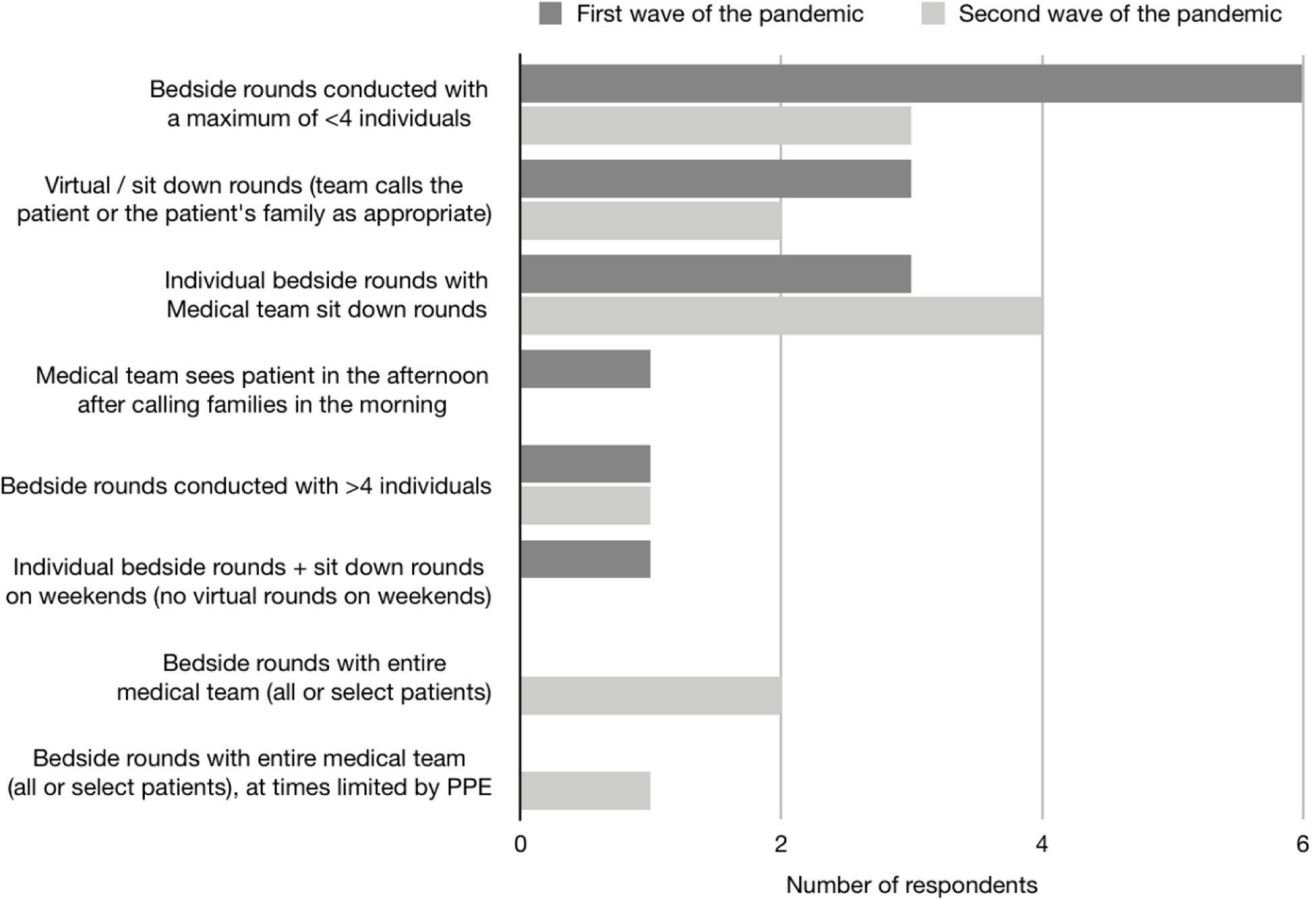
Survey respondents described how they would plan to implement CTUs after the first and second wave of the pandemic. The survey question was “After the first / second wave of the pandemic, how are you planning to implement CTUs?”

**Fig. 5:**
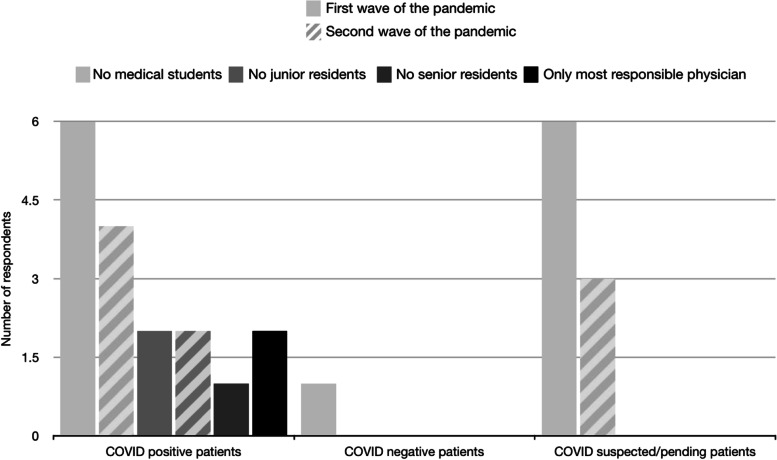
Respondents describe their plan for which team members are allowed to participate in the care of patients after the height of the first and the second wave of the pandemic. The survey question was “After the height of the first / second wave of the pandemic, which team members are allowed to participate in CTUs?”

After the height of the first wave of the COVID-19 pandemic, most hospitals (66.7%, *n* = 6) planned to conduct beside rounds with a maximum of four individuals. However, there was variability in the method for conducting rounds (Fig. 4). Most of the CTUs decided postgraduate learners were able to care for COVID positive or pending patients, however most respondents planned to continue to restrict medical students or in some cases only have the attending physician care directly for COVID-19 positive (7/9 respondents) or suspected patients (6/9 respondents) after the first wave of the pandemic (Fig. 5).

**Fig. 6:**
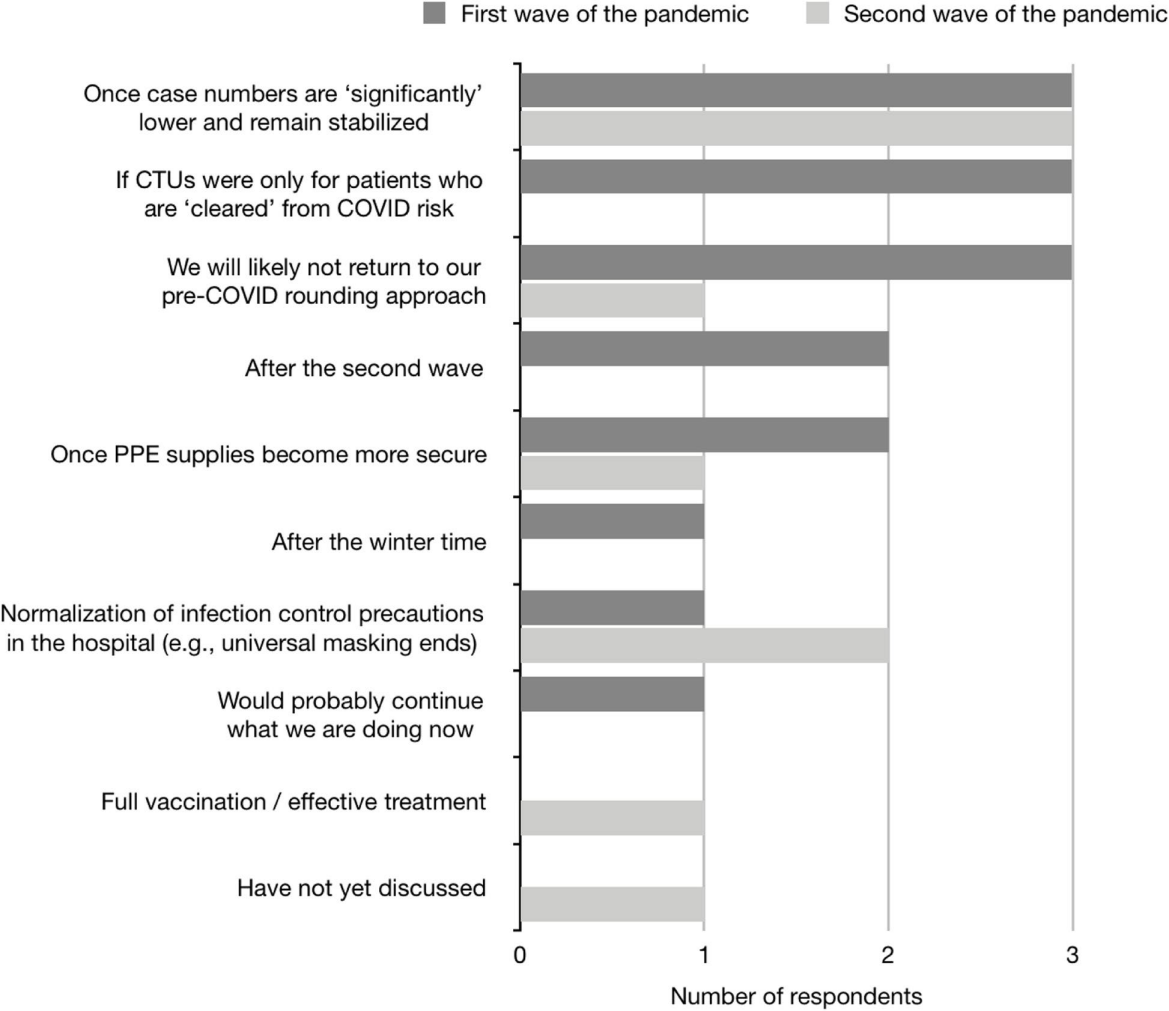
Survey question (“During the first / second wave of the pandemic, what would be required for CTU rounds to transition back to the pre-COVID method of rounding?”) and responses to the requirements for transitioning back to pre-COVID-19 method of CTU implementation during the first or second wave of the pandemic

There was inconsistency in the responses as to what would be required for CTU rounds to transition back to their pre-COVID-19 approach. A few hospital respondents selected that once COVID-19 case numbers are ‘significantly’ lower and remain stabilized or if CTUs were only for patients who are deemed COVID-19 negative, while others indicated they were unlikely to return to pre-COVID rounding indefinitely (Fig. 6).

### Second wave of the COVID-19 pandemic

To determine whether CTU implementation and planning have changed between the first and the second and more severe wave of the pandemic, we redistributed the survey in November 2020. We received eight responses (36% response rate) all representing pediatric tertiary hospitals and eight different medical schools (University of Ottawa, Université de Montréal, Dalhousie University, McMaster University, University of Toronto, University of Calgary, University of British Columbia, and McGill University). A majority (87.5%) of hospital CTU teams are responsible for 10-15 patients, and one hospital CTU team is responsible for 15-20 patients, similar to the first survey.

During the second wave of the pandemic, 50% (*n* = 4) and 37.5% (*n* = 3) of respondents were conducting bedside rounds with a maximum of 4 individuals or implementing individual bedside sit down rounds, respectively (Fig. 2), which is different from the first wave of the pandemic where individual bedside sit-down (*n* = 5, 55.6%) and virtual rounds (*n* = 4, 44.4%) was the most common approach. During the second wave of the pandemic, restrictions maintained for the care of patients on CTU were reduced as only medical students were restricted from COVID-19 suspected (50%, *n* = 4) and positive (62.5%, n = 5) patients, and junior residents were restricted from COVID-19 positive patients (25%, *n* = 2) (Fig. 3).

After the second wave of the pandemic, respondents planned to perform individual bedside rounds with medical team sit down rounds (50%, n = 4) or bedside rounds with less than 4 individuals (37.5%, *n* = 3) (Fig. 4), a significant reduction, compared to after the first wave of the pandemic where most respondents (66.7%, *n* = 6) were planning to conduct bedside rounds with less than 4 individuals. Planning for learner restrictions on CTU, in the future, after the second wave of the pandemic remained similar to during the second wave, with the exception of one respondent changing their answer to no learner restrictions (Fig. 5).

To transition back to the pre-COVID method of rounding, respondents required case numbers to be ‘significantly’ lowered and stabilized (37.5%, *n* = 3) or for the normalization of infection control precautions in the hospital (25%, *n* = 2) (Fig. 6). For this question, respondents from the first wave of the pandemic selected ‘after the second wave’ or ‘after the wintertime’, which were not selected by respondents during the second wave of the pandemic. In addition, one respondent required full vaccination or effective treatment to transition back to their pre-COVID method of rounding.

## Discussion

While there remains a lack of consensus on many aspects of CTU implementation before, during, and possibly after the COVID-19 pandemic, this study provides insights into the differing approaches to rounding on pediatric CTU in Canada.

As a consequence of the COVID-19 pandemic, few publications have discussed the implementation of rounding with results similar to what we found in our survey, e.g., the use of smaller groups, virtual video chat, telephone, and using call rooms [10, 13, 14, 16–18]. DeSanti et al. discuss patient- and family-centered video round implementation in their pediatric intensive care unit in Madison, USA. They developed standardized rounds using an iterative process involving a quality improvement team, nurses and other care providers. Some of their challenges included difficulty hearing presenters, frequent interruptions, scheduling team members, and compromised learner education. In a survey conducted in Pennsylvania, USA by Gaulton, et al.*,* 31% (7/49) of respondents answered that there are improvements to care as a result of virtual rounding, which included more efficient and shorter rounds, increased safety, and improved physical distancing [18]. The benefits of virtual rounding were expanded on by one respondent from our survey as they plan on maintaining a virtual process beyond the pandemic to improve care and communication for patients. While the majority of health care providers 98% (48/49) at this site felt that virtual rounding is safe, there was one concern addressing the improper assessment of a surgical patient [18]. They addressed this feedback to improve the accessibility of surgeons. This publication also elaborated on the benefits and shortcomings (e.g., technology difficulties, translator shortages) of different rounding methods. It may also be noteworthy that the survey respondents in the USA study were private or public hospitals, where clinical care may differ compared to our public health care system in Canada. Recommendations published by Frost et al. discuss Canadian guidance and considerations for conducting rounds on medical units, including the types of health care workers to be involved, methods of eliminating traffic and contact, using personal protective equipment observers/auditors, and advice for safely performing physical examinations [11]. However, the focus of this analysis was not specific to CTU rounds and they did not discuss the frequency, restrictions of learners, different rounding styles (e.g., virtual vs. sit-down), and the types of patients involved in rounds.

While there is discussion about the preparation for post-COVID-19 medical education [10, 19–21], there is no consensus about the threshold to returning or transitioning back to pre-COVID-19 rounding or implementation of CTUs in Canada. In a review of surveys about surgical mentorship, distance mentorship via telementoring was used to enhance surgical skill [13]. Aligning with our survey results, some believe that their medical education will not be returning to their pre-COVID-19 teaching platform, but towards a new normal [22]. Many programs, including participants of our survey, transitioned towards virtual and online synchronous and asynchronous learning platforms [3, 6, 14, 15]. Some challenges with virtual learning platforms include digital equity and technological literacy [3, 6]. The lack of physical connection and in-person contact can lead to the feeling of isolation [6]. It remains to be seen the impact on trainees in the long-term of not having the same degree of bedside teaching, an integral part of the learning environment in academic centers. These challenges were reflected by one respondent of our survey who elaborated on the difficulty of balancing patient care, pedagogic responsibilities towards learners, maintenance of a multidisciplinary care, and infection control precautions. A survey of pediatric residents in the United States during COVID-19 showed that remote learning and in-person sessions were similar in effectiveness and that they advocated for a hybrid model of teaching post-pandemic [7]. Supplemental approaches considered include simulation based learning, however the fidelity of these experiences in teaching many of the softer skills of clinical medicine remain unclear [23, 24].

Informed by the survey results, our Department of Pediatrics (McMaster Children’s Hospital) developed a new family-centered, virtual rounds process (Additional file 3). At the bedside, there will be a maximum of 5-6 people, including, the most responsible physician, subspecialty pediatric resident/fellow, nurse practitioner (NP), learner who has seen the patient, and the bedside nurse. The NP or designate will bring a tablet to virtually connect with other team members in the conference room. The conference room will be equipped with a computer to facilitate the virtual rounds and include the remaining team members and learners who are to physically distance. Consent will be required from patients/caregivers for virtual rounds.

### Limitations

An important limitation of this study is that the results are based on data collected from surveys [25] with low response rates (36-41%), thus these findings may not be generalizable and representative of the population. Low response rates attribute to some single results (i.e., *n* = 1) and while there is little support, it is still important to highlight the diversity and variance in responses across Canada. Due to the small sample size, it was meaningful to apply statistics as our study was not intended to have statistical power. Our survey respondents are CTU coordinators that are interested in this topic, thus may have biases and different perspectives from CTU participants. In addition, while survey respondents are expected to be representatives of their respective hospital, responses may be biased towards personal opinions and perspectives. Our responses in the first survey represent 4 of 17 (23.5%) Canadian schools and with the majority (*n* = 3) of the represented schools being in Ontario, Canada, the results are potentially limited to the provincial infection prevention and control guidelines of Ontario. In the second survey, there was representation from 8 of 17 Canadian schools (47.1%) (n = 3) from Ontario, (*n* = 2) from Québec, (*n* = 2) from Western provinces, and (*n* = 1) from Eastern provinces. From the respondent in Nova Scotia, they mentioned they have had a very low number of COVID-19 cases, which is why their CTU rounding practices only change when there are PPE shortages which results in a reduction of learners. Thus, our survey results are not limited to regional COVID-19 epidemiology, but also are impacted by other factors such as PPE availability. Since our survey respondents represent multiple areas of Canada, there is a risk of heterogeneity due to the differences in COVID-19 epidemiology across Canada. Lastly, while all four medical schools represented in the first survey also responded to the second survey, we had additional representation in our second survey which reduces the ability for direct comparisons over time and pandemic wave experiences between the first and second survey.

## Conclusions

These surveys were conducted for the general purposes of informing educational planning and policy development across CTU in Canada impacted by COVID-19. We realize there are differences in administration and local public health guidelines that impact implementation, thus these results should be considered taking into account regional differences. Our study highlights the variability in rounding practices during different stages of this pandemic and an opportunity to share approaches and lessons learned to optimize learner experience during unprecedent times for our academic hospitals. We anticipate further changes may be required if SARS-CoV-2 variants of concern require further restrictions, and may consider a subsequent survey should the need arise.

## Supplementary Information

**Additional file 1.**

**Additional file 2.**

**Additional file 3.**

## Data Availability

The datasets used and/or analysed during the current study available from the corresponding author on reasonable request.
