# Supplementary material for: A survey on Canadian pediatric hospital clinical/medical teaching unit implementation during the first and second wave of the COVID-19 pandemic
Source: BMC Med Educ. 2021 Nov 11;21:570. doi: 10.1186/s12909-021-02994-0 (PMC8580806; doi:10.1186/s12909-021-02994-0)
Supplement: Supplementary file 2 — Additional file 2. [file 12909_2021_2994_MOESM2_ESM.docx]

**Additional file 2:** Second survey on Canadian pediatric clinical teaching unit implementation distributed between November-December 2020

**
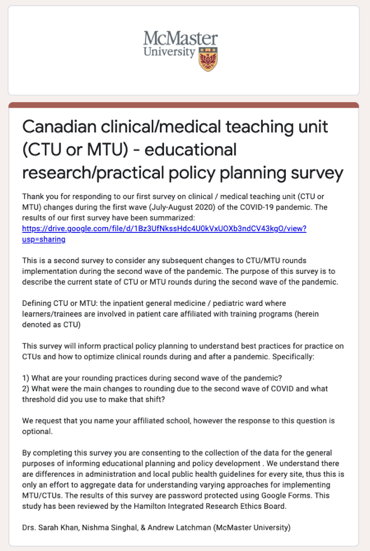
**

**
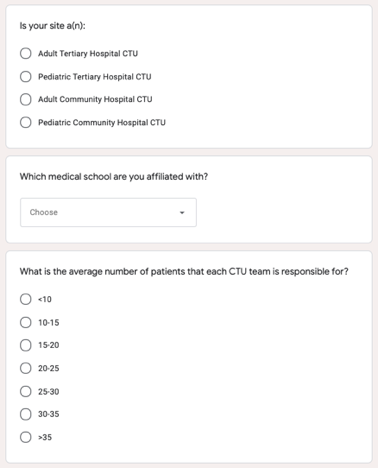
**

**
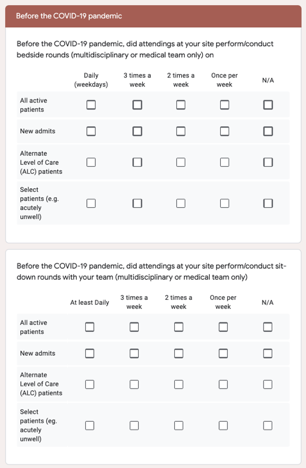
**

**
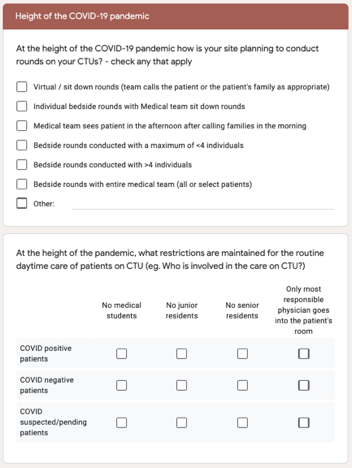
**

**
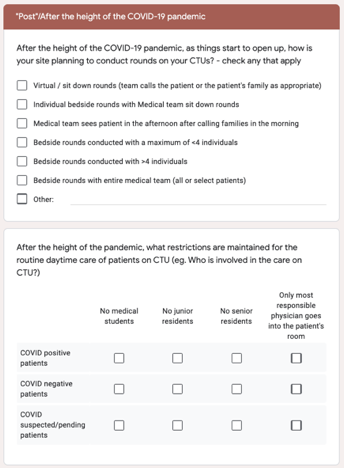
**

**
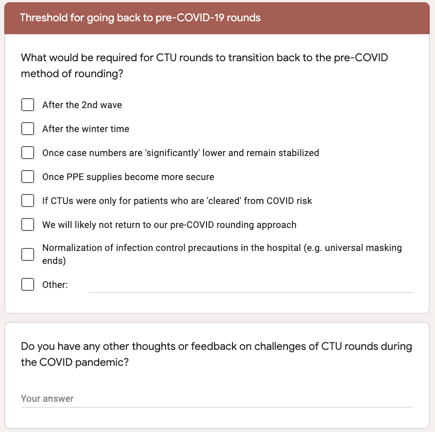
**
