# Supplementary material for: A survey on Canadian pediatric hospital clinical/medical teaching unit implementation during the first and second wave of the COVID-19 pandemic
Source: BMC Med Educ. 2021 Nov 11;21:570. doi: 10.1186/s12909-021-02994-0 (PMC8580806; doi:10.1186/s12909-021-02994-0)
Supplement: Supplementary file 3 — Additional file 3. [file 12909_2021_2994_MOESM3_ESM.docx]

**Additional file 3**

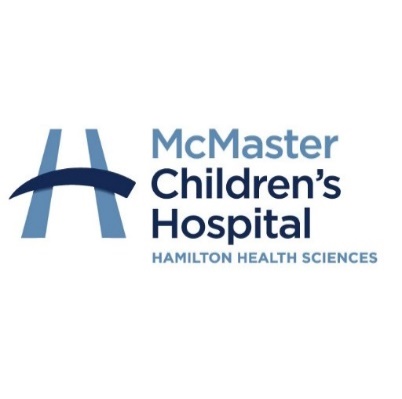


**McMaster Children’s Hospital – CTU Pediatric Medicine**

**Virtual Family Centred Rounds for COVID-19**

**NEW process for family-centred rounds:** Virtual Rounds via HHS Zoom with iPads

- Limited number of people at bedside, with remaining participants joining virtually

**Go Live:** January 4, 2021

Virtual Rounds Process

NPs will set up a HHS Zoom session each day for CTU rounds.

Charge Nurse will still create daily rounding schedule to facilitate nurse attendance.

**Bedside:** 5-6 people

- MRP, SPR/fellow, NP, learner who has seen the patient, bedside nurse
- NP or designate will bring iPad to virtually connect with other team members not in room
  - iPad to share video and audio of bedside rounds to Virtual team members
  - iPad to show video from Virtual team members in conference room

**3C or 3Z conference room:** remaining team members and learners

- Team 1 and Team 2 will each home base from one conference room (3C or 3Z)
- COW (computer) in each conference room is equipped with Zoom, video camera, and speaker to participate in virtual rounds
  - 3Z: COW is connected to TV
  - 3C: COW screen will have Zoom session; TV is pending installation

**Consent** is required from patients/caregivers for virtual care (see current forms on next page)

- Information sheet about Virtual Rounding will be laminated and on the wall in each room.
- Consent forms will be available on each rounding cart.
- Consent forms will need to be completed for each patient prior to Virtual Rounds and placed in front of chart for remainder of admission.
- If a patient/caregiver does not consent for virtual rounds, iPad camera and sound will be switched off/muted for that patient, and learners in conference room can do rounds challenge in that time.

**Other notes:**

- iPads must be sanitized between patients (wipes for electronics on each rounding cart)
- in conference room, camera should ideally be positioned to capture all attendees
- in conference room, staff and learners must maintain physical distancing and room capacity limitations (3C - 6 people, 3Z - 10 people).

Information Sheet - Virtual Rounding in Pediatric Medicine

Protecting Patients and Staff During COVID-19

**What is a virtual rounding and why are we using it?**

On our Pediatric Medicine unit, the medical and nursing team meet with families Monday to Friday mornings to create a plan of care for the patient. McMaster Children’s Hospital is a teaching hospital, with many learners being part of the team that delivers patient care. As such, the rounding group can be quite large. In an effort to protect patients and staff during the COVID-19 pandemic we are modifying our rounds so that only a few members of the healthcare team will come to the patient’s bedside and the rest of the team will visit virtually using a zoom call from our unit’s conference room.

**Understanding privacy and security**

Our conference room is set up with a computer, and this will be the only device connected to your rounding session. All of the people in the room are part of the team caring for your child. There is a closed door which will help to ensure the information shared cannot be heard by others. Since we will be using Zoom for the virtual rounds, it is important for you to understand that we are taking all reasonable steps to protect the privacy of your information, but it is still possible that your information may be routed through the U.S.A. It is not recorded, stored or otherwise retained there.

Consent - Virtual Rounding in Pediatric Medicine


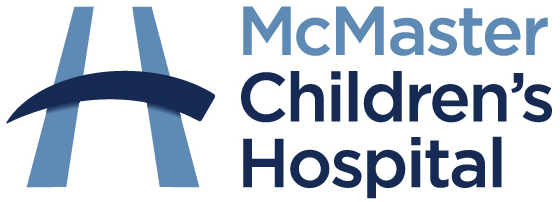


Bradma

Informed verbal consent was obtained from this patient and/or parent/substitute decision maker to communicate and provide care using virtual rounding. The risks related to unauthorized disclosure or interception of personal health information have been explained.

__________________________ _________________________ _____________

Printed Name of Healthcare Provider Signature Date

Source: “Introduction of Fee Code for Physicians Providing Services by Telephone or Video or Working in Designated Assessment Centres,” Ontario Medical Association, March 13, 2020
